# Highly effective proximate labeling in *Drosophila*

**DOI:** 10.1093/g3journal/jkab077

**Published:** 2021-03-16

**Authors:** Bo Zhang, Yuanbing Zhang, Ji-Long Liu

**Affiliations:** 1 School of Life Science and Technology, ShanghaiTech University, Shanghai 201210, China; 2 Institute of Biochemistry and Cell Biology, Shanghai Institutes for Biological Sciences, Chinese Academy of Sciences, Shanghai 200031, China; 3 University of Chinese Academy of Sciences, Beijing 100049, China; 4 Department of Physiology, Anatomy and Genetics, University of Oxford, Oxford OX1 3PT, UK

**Keywords:** protein–protein interaction, TurboID, proximate labeling, CTP synthase, cytoophidium, mass spectrometry, *Drosophila*

## Abstract

The protein–protein interaction (PPI) is a basic strategy for life to operate. The analysis of PPIs in multicellular organisms is very important but extremely challenging because PPIs are particularly dynamic and variable among different development stages, tissues, cells, and even organelles. Therefore, understanding PPI needs a good resolution of time and space. More importantly, understanding *in vivo* PPI needs to be realized *in situ*. Proximity-based biotinylation combined with mass spectrometry (MS) has emerged as a powerful approach to study PPI networks and protein subcellular compartmentation. TurboID, the newly engineered promiscuous ligase, has been reported to label proximate proteins effectively in various species. In *Drosophila*, we systematically apply TurboID-mediated biotinylation in a wide range of developmental stages and tissues, and demonstrate the feasibility of TurboID-mediated labeling system in desired cell types. For a proof-of-principle, we use the TurboID-mediated biotinylation coupled with MS to distinguish CTP synthase with or without the ability to form filamentous cytoophidia, retrieving two distinct sets of proximate proteomes. Therefore, this makes it possible to map PPIs *in vivo* and *in situ* at a defined spatiotemporal resolution, and demonstrates a referable resource for cytoophidium proteome in *Drosophila*.

## Introduction

The interaction between protein and protein serves as a basic strategy for various complex biological processes (BP). Much of our fundamental knowledge about protein–protein interactions (PPIs) comes from traditional biochemical methods, which mostly capture *in vitro* snapshots. However, PPIs are highly dynamic, especially for multicellular organisms. In different stages of development, different organs and tissues, cells and even organelles and other structures in cells, the PPI will have great changes.

We are eager to develop new tools to explore PPIs, at least to meet the following four requirements. First, it must be *in vivo*, not *in vitro*, to ensure that it reflects the living physiological condition. Second, it needs to be *in situ* to capture the relationship between proteins without interference. Third, there needs to be a good enough time window to understand special PPI in controllable developmental stages. Fourth, it can identify the PPI of a certain organ, tissue, cell type, organelle, and even different forms of organelles. It is very challenging to meet all of the above four requirements.

Recently, Ting and colleagues directly evolved the *E. coli* biotin ligase BirA using yeast display and generated two promiscuous labeling variants, TurboID and miniTurbo, which enable sufficient proximity labeling in just 10 min with the use of nontoxic biotin (Branon *et al.* 2018). TurboID and miniTurbo have been demonstrated to probe different organellar proteomes in HEK cells (Branon *et al.* 2018). Because of the high labeling efficiency and lower temperature requirement, TurboID and miniTurbo have been utilized to profile interaction networks in *S. pombe*, *N. benthamiana*, and *Arabidopsis* (Larochelle *et al.* 2019; Mair *et al.* 2019; Zhang *et al.* 2019).

CTP synthase (CTPS), an essential metabolic enzyme responsible for the *de novo* synthesis of nucleotide cytidine triphosphate (CTP), has been shown to form filamentous structures in *Drosophila (*Liu 2010*)*, bacteria (Ingerson-Mahar *et al.* 2010), and budding yeast (Noree *et al.* 2010). These filamentous structures have been referred to as cytoophidia (Greek for “cellular serpents”) (Liu 2010). Subsequently, cytoophidia were found in mammalian cells (Carcamo *et al.* 2011; Chen *et al.* 2011), fission yeast (Zhang *et al.* 2014), plants (Daumann *et al.* 2018), and archaea (Zhou *et al.* 2020), which indicates their evolutionary conservation. CTPS catalyzes the ATP-dependent transfer of nitrogen from glutamine to UTP, forming glutamate and CTP (Anderson 1983; Koshland and Levitzki 1974; Weng and Zalkin 1987). The product of CTPS catalytic reaction, CTP, not only serves as an essential nucleotide and precursor for the synthesis of RNA and DNA, but also participates in the membrane phospholipid synthesis and protein sialylation (Liu 2016).

However, the mechanisms of how CTPS cytoophidia affect CTPS activity and control CTP levels are poorly understood. CTPS cytoophidia are dynamic during *Drosophila* development and exhibit heterogeneous distribution in many tissues (Zhang *et al.* 2020), and appear in various human cancers (Chang *et al.* 2017). A previous study has indicated that CTPS is critical for brain development and optic lobe homeostasis in *Drosophila* (Tastan and Liu 2015). We have recently reported that TOR pathway could modulate CTPS cytoophidia assembly in mammalian cells (Sun and Liu 2019b) and fission yeast (Andreadis *et al.* 2019). In bacteria, CTPS polymerization is essential for cellular homeostasis and cell shape maintenance (Ingerson-Mahar *et al.* 2010). Recently, we demonstrated that forming filaments could inhibit CTPS ubiquitination and further prolong the half-life of CTPS, indicating that CTPS cytoophidium may serve as a metabolic stabilizer in cells (Sun and Liu 2019a). The assembly or elongation of cytoophidia are affected by nutritional condition (Aughey *et al.* 2014; Noree *et al.* 2010), transcriptional factor Myc (Aughey *et al.* 2016), E3 ligase Cbl (Pai *et al.* 2016), Ack kinase (Strochlic *et al.* 2014). Cytoophidia assembly facilitates enzymatic regulation (Barry *et al.* 2014; Lynch *et al.* 2017). Other roles of CTPS cytoophidia, such as in developmental switch, stress coping, and intracellular transport, have been proposed, but not yet studied (Liu 2016). Characterization of the proteome in subcellular compartments is essential for the identification of protein interaction networks and the understanding of organelle organization as well as of complex BP. However, CTPS cytoophidium proteome has not yet been mapped spatiotemporally in any species* in vivo*.

Ting and colleagues also used the proximite labeling ability of TurboID and miniTurbo in *Drosophila* and *C. elegans* systems (Branon *et al.* 2018). In *Drosophila*, their work was to construct the transgenic flies expressing TurboID and miniTurbo, which was proved by Western blotting that TurboID/miniTurbo could biotinylate proteins effectively in *Drosophila*. However, there are still a few questions that have not been answered clearly. First, they did not have fused bait proteins. Second, there was no testing to see if TurboID and miniTurbo interfered with the location of the bait protein *in vivo*. Finally, they did not further use mass spectrometry (MS) to test whether TurboID and miniTurbo can label neighboring proteins *in vivo*.

Based on the background, we try to answer these following questions. (1) Will the localization of the bait protein fused by TurboID or miniTurbo be interfered? (2) Can TurboID or miniTurbo be used to fuse bait proteins *in vivo*? (3) Can we make proximity labeling *in situ*? (4) If the distribution of the bait protein is disturbed, can different binding proteins be obtained?

Here, we use the TurboID-mediated biotinylation coupled with MS method to address those questions. For a proof-of-principle study, we use CTPS as an example. An H355A point mutation of CTPS can disrupt its cytoophidium-forming ability (Lynch *et al.* 2017; Sun and Liu 2019a; Zhou *et al.* 2019), which provides an excellent negative control. We apply TurboID-mediated labeling to a variety of developmental stages and tissues in *Drosophila*. Using a cell-specific GAL4 driver, we verify that TurboID can biotinylate the bait protein CTPS, making possible the identification of PPIs in individual cells. Using the wild-type and mutant CTPS as bait proteins, these results in two distinct sets of proximate proteomes. Identification of cytoophidia proteomic networks facilitates the understanding of its organization as well as BP. Our results suggest that TurboID-mediated labeling system is a feasible tool to catch *in vivo* PPIs *in situ* at a defined spatiotemporal resolution.

## Materials and methods

### Construction of plasmids

*Drosophila* codon-optimized TurboID and miniTurbo sequences were synthesized. The sequences of CTPS, Catsup, and Pdcd4 were obtained from *Drosophila* cDNA. These sequences containing epitope tag were amplified by PCR (Vazyme, Cat. # P505-d3). pAc 5.1 vector was digested by EcoRI and NotI(NEB). The amplified CTPS, TurboID-V5, and miniTurbo-V5 were inserted into pAc vector by seamless cloning (Vazyme, Cat. # C113-02) to result in plasmids pAc 5.1 CTPS-TurboID-V5 and pAc 5.1 CTPS-miniTurbo-V5. In pAc-CTPS-HA-T2A-Catsup-V5 and pAc-CTPS-HA-T2A-Pdcd4-V5, CTPS-HA and Catsup-V5 or Pdcd4-V5 can be produced from one transcript using self-cleaving T2A. To induce H355A directed point mutation in CTPS, the following primers were used to amplify wild-type CTPS encoding sequence:

F: 5′-GAGCAAGTACGCCAAGGAGTGGCAGAAGCTATGCGATAGCCA-3′

R: 5′-CACTCCTTGGCGTACTTGCTCGGCTCAGAATGCAAAGTTTCC-3′

To create pUASt-CTPS-mCherry-V5, pUASt-CTPS-TurboID-V5, and pUASt-CTPS^H355A^-TurboID-V5 plasmids, mCherry-V5, TurboID-V5, and CTPS^H355A^-V5 were amplified and inserted into pUASt vector by seamless cloning. pUASt attB vector was digested using NotI and KpnI (NEB). The final constructs were sequenced before injection.

### Cell culture

S2 cell line was maintained in Schneider’s *Drosophila* medium (Gibco) supplemented with 10% fetal bovine serum at 28°C incubator. Cell transfections were carried out using Effectene Transfection Reagent (QIAGEN) according to manufacturer’s instructions.

### Transgenic flies and *Drosophila* culture

Three transgenic fly lines were established in our study. Constructs with the attB sequence were injected into fly germline (attP2) using PhiC31 integrase-mediated site-specific integration, which was carried out by co-injection with phiC31 integrase RNA at the Core Facility of *Drosophila* Resource and Technology, Institute of Biochemistry and Cell Biology, Shanghai Institutes for Biological Sciences, Chinese Academy of Sciences. These transgenes are integrated in chromosomes. To express ligase TurboID ubiquitously or germ-cell specifically in flies, transgenic flies were crossed with da-GAL4 or nanos-GAL4 driver flies and recombinants were generated. All flies were raised at 25 °C on either standard cornmeal food or 100 μM biotin-containing food accordingly.

### Immunofluorescence

For S2 cells, after 36-hours transfection, cells cultured on glass slides were fixed with 4% (w/v) paraformaldehyde in PBS for 20 min. Cells were washed three times with PBS and then permeabilized with 0.2% Triton X-100 for 15 min. After blocking with 5% (w/v) bovine serum albumin (BSA) in PBS for 1 hour, primary antibody incubation with anti-V5 antibody (1:500, Invitrogen, Cat. # 460705) in PBS was carried out overnight at 4 °C. Following three washes in PBS, cells were incubated with Alexa Fluor 488-labeled secondary antibody (1:500, Invitrogen, Cat. # A11029) and Hoechst 33342 (1:10000, Bio-Rad, Cat. # 151304) for 1 hour.

For *Drosophila*, ovaries from 14-day flies were dissected in Grace’s Insect Medium (Life) and then fixed with 4% (w/v) paraformaldehyde in PBS for 20 min. Ovaries were incubated with anti-V5 antibody (Invitrogen) in PBS containing 0.3% Triton X-100 and 0.5% horse serum overnight. To detect the distribution of biotinylated proteins and expression of ligase, ovaries were incubated with Alexa Fluor 488-labeled secondary antibody (1:500, Thermo Fisher) and Streptavidin-Cy3 (1:300, Jackson, Cat. # 016-160-084) containing Hoechst 33342 (Bio-Rad) overnight before confocal imaging.

### Protein extraction and western blotting

To extract proteins from flies of different developmental stages, larvae, pupae, and adult flies were collected and frozen with liquid nitrogen for 1 min. For protein extraction from different tissues, the tissues were dissected in Grace’s Insect Medium (Life) and then were frozen with liquid nitrogen for 1 min. Samples were prepared with RIPA lysis buffer and 1X protease inhibitor cocktail (Bimake) and then ground for 10 min. Following 20 min incubation on ice, samples were centrifuged at 13,000 rpm at 4 °C for 30 min. The supernatants were collected and boiled with 1X protein loading buffer at 95 °C for 10 min. Lysates were separated by 4–20% SDS-PAGE gels, followed by transferring to PVDF membranes (Roche). After blocking with 5% BSA in TBST for 1 hour, membranes were incubated with HRP-conjugated antibody (1:3000, Cell Signaling, Cat. # 3999 s) for 1 hour. After three times washing in TBST, biotinylated proteins were visualized using the enhanced chemiluminescence system AI600 (GE). To detect the recombination expression of ligase, blocked membranes were incubated with anti-V5 antibody (1:3000, Invitrogen) at 4 °C overnight. Following three washes in TBST, membranes were incubated with HRP-linked anti-mouse antibody (1:3000, Cell Signaling, Cat. # 7076 s) for 1 hour. Then, membranes were washed with TBST three times and visualized by the enhanced chemiluminescence system.

### Immunoprecipitation

*Drosophila* S2 Cells were harvested 24 hours after transfection, and were lysed in Lysis buffer (20 mM Tris-HCl, pH 7.4, 50 mM NaCl, 2 mM MgCl2, 1% [vol/vol] NP40, 0.5% [mass/vol] sodium deoxycholate, and 0.1% [mass/vol] sodium dodecyl sulfate) containing 1X protease inhibitor cocktail (Bimake) for 2 hours at 4 °C. After being centrifuged at 15,000 rpm for 10 min, the supernatant was incubated with anti HA magnetic beads and IgG bound Protein A/G magnetic beads (Bimake) equally and gently agitated sample overnight at 4 °C. Then the beads were washed three times with 1 ml PBST (0.5% Tween20 in PBS) wash buffer to remove nonspecific binding. Finally, 50 μl 1 × loading buffer containing SDS was added into each sample and the interaction was detected by immunoblotting. Immunoprecipitation and western blotting analyses were performed as indicated three times with similar results.

### MS sample preparation

About 60 ovaries from 14-day-old adult flies grown on 100 μM biotin-containing food were dissected, then fixed with 4% (w/v) paraformaldehyde in PBS for 20 min, followed by washing with cold PBS one time, and then incubated with 1 ml lysis buffer at 4 °C. After shaking for 1 hour, the lysate was spun down at 4 °C for 10 min. The supernatant was transferred into new tubes, with the addition of urea and DTT to a final concentration of 8 M and 10 mM. The lysate was incubated at 56 °C for 1 hour, then treated with 25 mM iodoacetamide in the dark for 45 min to aminocarbonyl modify the Cys site of proteins. 25 mM DTT was added to terminate the modification. 50 μl Streptavidin Magnetic Beads (NEB, Cat. # S1420S) were washed with 500 μl PBS three times and then resuspended into the lysate. Subsequently, the lysate along with 50 μl beads was incubated on a rotator at 4 °C overnight. The beads were washed with the following buffers: twice with buffer 1 (50 mM Tris8.0, 8 M urea, 200 mM NaCl, 0.2% SDS), once with buffer 2 (50 mM Tris8.0, 200 mM NaCl, 8 M urea), twice with buffer 3 (50 mM Tris8.0, 0.5 mM EDTA, 1 mM DTT), three times with buffer 4 (100 mM ammonium carboxylate), and finally the beads were resuspended in 100 μl buffer 4. Trypsin, 4 μg (Promega, Cat. # v5113) was added to digest proteins to generate peptides overnight at 37 °C. The peptides were collected with ziptip by the addition of 1% formic acid, then washed with 0.1% TFA (Sigma, Cat. # 14264) and eluted in 50 μl of 70% ACN (Merck Chemicals, Cat. # 100030)-0.1% TFA. The peptides were analyzed on an Orbitrap Fusion.

### MS data analysis

The UniProt *Drosophila* melanogaster protein database (Proteome ID: UP000000803), and database for proteomics contaminants from MaxQuant were used for database searches. Reversed database searches were used to evaluate the false discovery rate (FDR) of peptide and protein identifications. Two missed cleavage sites of trypsin were allowed. The FDR of both peptide characterization and protein characterization was set to be 1%. The options of “Second peptides,” “Match between runs,” and “Dependent peptides” were enabled. For differential expression analysis, the limma-based approach evolutionarily in R 3.6.1 was used. The data were log2 transformed and centered, and the statistical significance of the biological repeats of CTPS cytoophidium and disrupted cytoophidium control was tested using a modified *t*-test in limma 3.40.0. Enriched proteins of CTPS cytoophidium with fold change >1.5 and *P* < 0.05 were defined as up-regulated proteins and those with fold change <0.67 and *P* < 0.05 were defined as down-regulated proteins. Functional enrichment analysis was followed to define the enriched proteins of CTPS cytoophidium. clusterProfiler software was used to obtain enriched GO terms corresponding to biological processes (BPs), cellular components (CCs), and molecular functions (MFs) (Yu *et al.* 2012). Only those categories with *P*-adjust lower than 0.05 were considered to be reliable. Previously reported CTPS-interacting proteins were obtained from STRING database (https://string-db.org, last accessed on March19, 2021).

### Data availability

Strains and plasmids are available upon request. Supplemental material is available at figshare: https://doi.org/10.25387/g3.14067707. 

## Results

### Will the localization of the bait protein fused by TurboID or miniTurbo be interfered?

**Figure 1:**
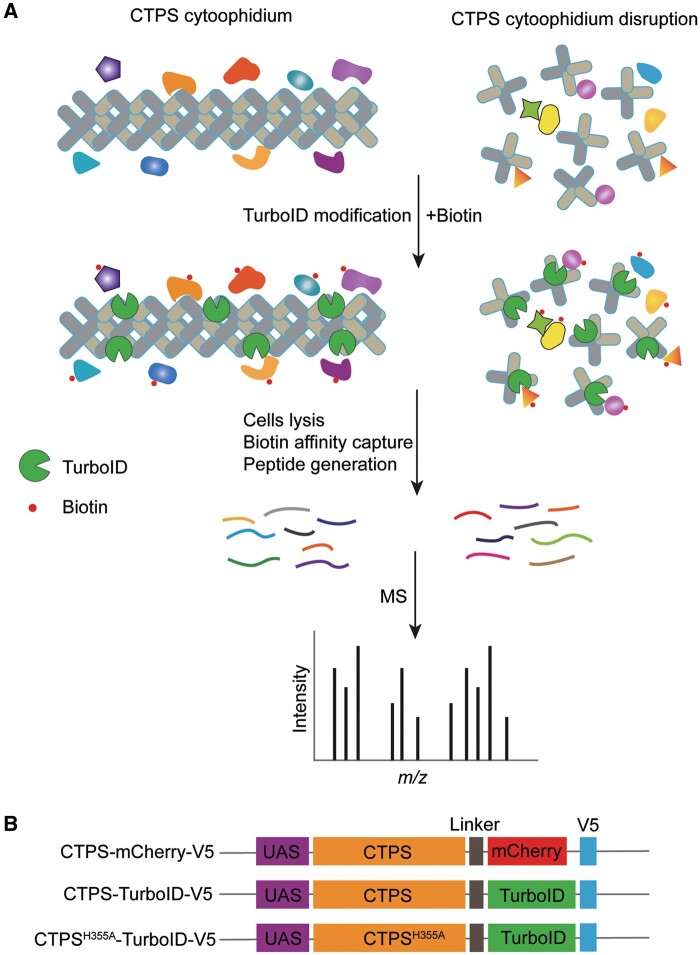
A design of TurboID-mediated proximity labeling method to map CTPS proximate proteomes*.* (A) TurboID was fused in-frame with wild-type and mutant CTPS. Provided with biotin, TurboID can use biotin to biotinylate CTPS neighboring proteins. Cells are lysed and biotinylated proteins are captured using streptavidin beads. Subsequently, small peptides are generated by trypsin digestion and peptides are analyzed by MS. Note that just a finite number of TurboID are shown in CTPS cytoophidium and disrupted cytoophidium. (B) Diagram of the expression cassettes used for the generation of transgenic flies. TurboID and V5 tag were fused to the C-terminal of wild-type and mutant CTPS. mCherry was used as control. The same flexible linker was inserted into three cassettes.

**Figure 2:**
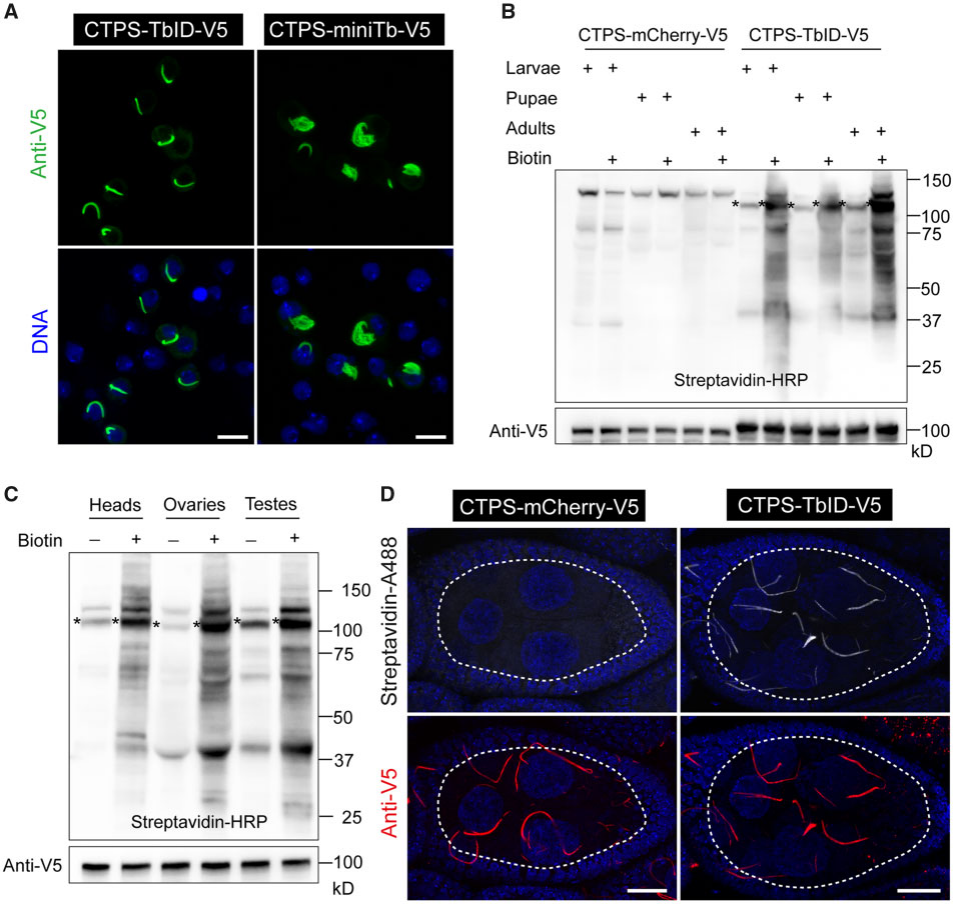
TurboID application in *Drosophila*. (A) CTPS-TbID and CTPS-miniTb containing V5 tag at C-terminal were cloned into pAc vectors and were transfected into *Drosophila* cultured S2 cells. Confocal images of cells are presented. DNA was labeled with Hoechst 33342 (blue). Scale bar, 10 μm. (B) Transgenic flies of UAS-CTPS-mCherry-V5 and UAS-CTPS-TbID-V5 were generated. Flies raised on either biotin-containing food or regular food from early embryo stages to larvae, pupae or adulthood, were collected and lysed and then blotted with Streptavidin-HRP to visualize biotinylated proteins. Anti-V5 antibody was used to detect the fused expression of CTPS-mCherry and CTPS-TbID, which was expressed ubiquitously via da-GAL4 driver. The molecular weight of CTPS-mCherry (98 kD) is a little smaller than CTPS-TbID (105 kD). Star (*) indicates the location of biotinylated CTPS-TbID-V5. (C) CTPS-TbID was expressed ubiquitously via da-GAL4 driver. Western blotting with streptavidin-HRP to visualize biotinylated proteins in different tissues from adult flies raised on either 100 μM biotin-containing food or regular food is presented. CTPS expression was detected by anti-V5 blotting. Star (*) indicates the location of biotinylated CTPS-TbID-V5. (D) Both CTPS-mCherry and CTPS-TbID were specifically expressed in germline cells driven by nanos-GAL4. Outlined areas show large nurse cells surrounded by monolayer follicle cells. Ovaries from 14-day-old flies grown on 100 μM biotin-containing food were dissected. Representative images of biotinylated proteins were obtained after detection by staining with streptavidin-488, while the expression of CTPS-mCherry and CTP-TbID was detected by anti-V5 blotting. Scale bar, 20 μm.

To map the proteome of CTPS cytoophidium in *Drosophila*, we designed the workflow (Figure 1A). And the initial concern we had to address was that the filamentous structures of CTPS should not be affected under recombinant expression with TurboID or miniTurbo (hereafter called TbID or miniTb). We checked for any conformational changes on CTPS cytoophidia by fusing TbID or miniTb containing V5 tag to the C-terminal of CTPS followed by transfection into *Drosophila* cultured S2 cells. Immunofluorescence results showed that CTPS retained its filamentous structures when fused with TbID, while the conformation of CTPS tagged with miniTb became irregular and filaments were disrupted (Figure 2A). Branon *et al.* (2018) reported that miniTb is less stable than TbID likely due to the removal of its N-terminal domain, which may explain that miniTb disrupted CTPS filamentous structures in *Drosophila* cultured cells. Considering the effects on CTPS cytoophidium conformation, we chose TbID as a promiscuous ligase in our study.

### Can TurboID be used to fuse bait proteins *in vivo*?

To demonstrate TbID proximate labeling feasibility of CTPS in *Drosophila*, we initially generated UAS-CTPS-TbID and UAS-CTPS-mCherry transgenic flies, containing V5 tag at C-terminal of recombinant protein (Figure 1B). We then examined TbID wider application in all growth stages in *Drosophila*, and, to do so, we used da-GAL4 to drive CTPS-TbID and CTPS-mCherry ubiquitous expression. Here, UAS-CTPS-mCherry served as a control group (Figure 2B). Flies, raised on either biotin-containing food or regular food from early embryo stages to larvae, pupae, or adulthood, were collected and lysed. Streptavidin-HRP blotting results indicated that TbID biotinylated proteins in a wide variety of developmental stages in the presence of exogenous biotin, while very few labeling signals were detectable in any stages in the CTPS-mCherry group and CTPS-TbID control group (Figure 2B).

We then dissected some tissues from adult flies in which CTPS-TbID and CTPS-mCherry were expressed via da-GAL4 driver. Western-blotting results using streptavidin-HRP revealed that proteins are extensively biotinylated in heads, ovaries, and testes with biotin feeding (Figure 2C and Supplementary Figure S1). Recently, Shinoda *et al.* (2019) knocked *TbID* gene into the C-terminal domain of caspase proteins’ gene loci by utilizing CRISPR/Cas9 technology and labeled potential neighboring proteins in wings. In agreement with these studies, our results show that TbID can be used to label endogenous proteins in a wide range of developmental stages and desired tissues in *Drosophila*.

### Can we make proximity labeling *in situ*?

After verifying the general applicability of TbID to proximity labeling in *Drosophila*, we wanted to further test whether TbID could biotinylate proteins and characterize local proteomes in individual cell types in *Drosophila*. A typical ovary in adult flies contains 16 ovarioles, each being tipped with the germarium which is followed by the growing egg chambers (Liu 2010; Spradling 1993). Each egg chamber includes three major cell types: one oocyte, 15 nurse cells, and hundreds of follicle cells (Spradling 1993). CTPS has been reported to form filaments in all three major cell types in ovaries (Liu 2010; Wu and Liu 2019).

Here, we used nanos-GAL4, a driver controlling gene expression in germline stem cells and spermatogonia, to achieve CTPS direct expression in nurse cells and oocytes, but not in follicle cells in ovaries. We then dissected ovaries from 14-day-old flies grown on biotin-containing food. Using anti-V5 antibody, we found that CTPS-mCherry and CTPS-TbID were both expressed and formed long and curved filamentous structures in nurse cells, whereas they did not in surrounding follicle cells, as expected (Figure 2D). The morphology of CTPS cytoophidia in nurse cells was similar to previous studies (Azzam and Liu 2013).

In CTPS-mCherry group, no obvious labeling signals were detected after staining with streptavidin-AlexaFluor488 (Figure 2D). In contrast, in the case of CTPS-TbID, immunofluorescence images revealed that the biotinylated proteins are characterized by extensive signals and form patterns almost identical to CTPS cytoophidia (Figure 2D), indicating that the proteins in the vicinity of CTPS were preferentially biotinylated by TbID. Thus, our study shows that the TbID-based proximity labeling system can be successfully used in labeling neighboring proteins of interest in desired cells using a cell-type-specific driver in *Drosophila*.

### If the distribution of the bait protein is disturbed, can different binding proteins be obtained?

**Figure 3:**
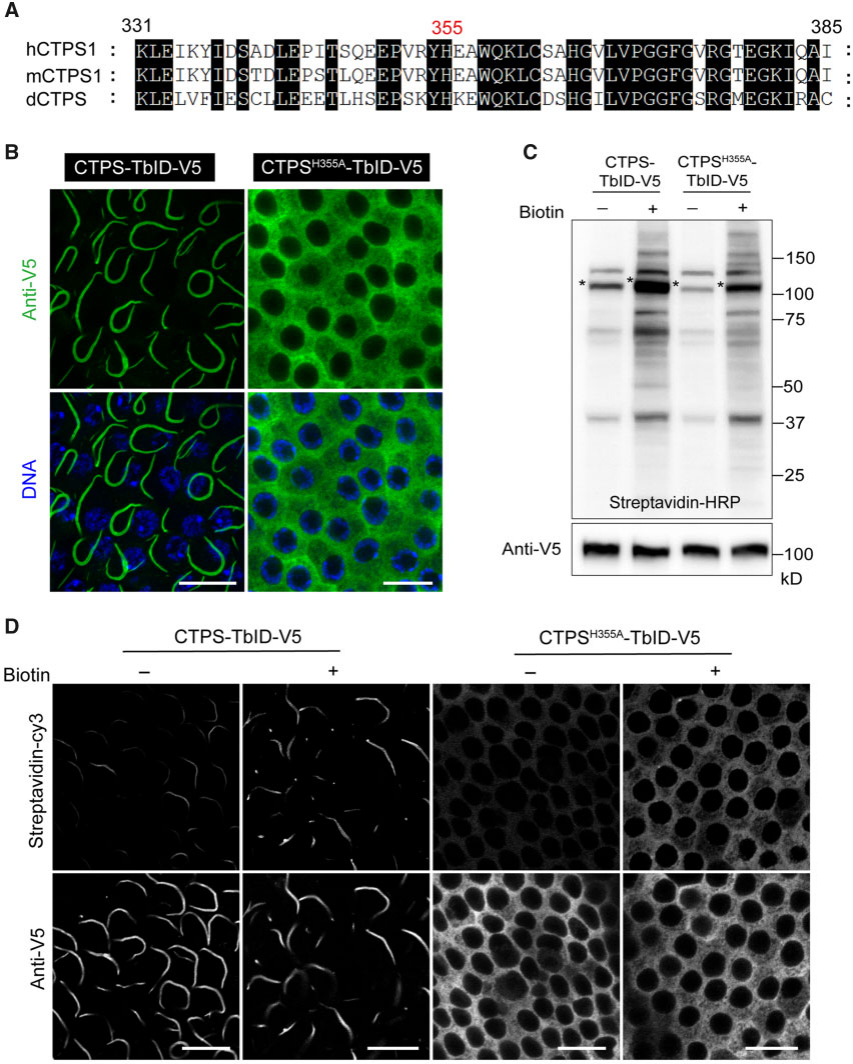
Proximity labeling of CTPS cytoophidium and mutant CTPS. (A) Amino acid sequence alignment among hCTPS, mCTPS, and dCTPS. A partial view is presented here. (B) Immunostaining results of CTPS-TbID and CTPS^H355A^-TbID are shown in follicle cells. Scale bar, 10 μm. (C) Streptavidin-HRP was used for the detection of labeled proteins, while anti-V5 antibody was used to detect the expression of CTPS-TbID and CTPS^H355A^-TbID. Star (*) indicates the location of biotinylated CTPS-TbID and CTPS^H355A^-TbID. (D) Ovaries were dissected from 14-day-old flies and raised on either 100 μM biotin-containing food or regular food. Confocal images of labeled proteins detected by staining with streptavidin-Cy3 are presented, along with the expression of CTPS-TbID and CTPS^H355A^ -TbID detected by anti-V5 blotting. All images were acquired from follicle cells. Scale bar, 10 μm. All ovaries samples were collected from 14-day-old flies and CTPS-TbID and CTPS^H355A^-TbID were expressed using da-GAL4 driver in (B–D).

A single histidine mutation on the tetramerization interface, H355A, renders hCTPS1 unable to polymerize into filaments in the presence of substrates (Lynch *et al.* 2017). Sun *et al.* have confirmed that the mutation H355A on mouse CTP synthase 1 (mCTPS1) also disrupts mCTPS1 assembly in mammalian cells (Sun and Liu 2019a). The amino acid histidine 355 (H355) was conserved among hCTPS1, mCTPS1, and dCTPS (Figure 3A). In order to characterize the proteome of CTPS cytoophidium, we worked in parallel with two distinct groups: CTPS formed filamentous structures in one group, while CTPS cytoophidia were disrupted in the control group.

To detect whether H355A mutation impedes CTPS cytoophidium assembly in *Drosophila*, we generated a UAS-CTPS^H355A^-TbID transgenic fly by fusing a V5 tag at its C-terminal (Figure 1B). CTPS-TbID and CTPS^H355A^-TbID were both expressed by da-GAL4 driver, and ovaries from adult flies were used to examine the conformation of CTPS. Immunofluorescence results revealed that wild-type CTPS formed long and curved filaments in follicle cells, while mutant CTPS showed a completely diffused distribution in cells (Figure 3B). These results indicated that H355A mutation disrupts CTPS polymerization in *Drosophila* follicle cells, providing an ideal control to characterize the neighboring proteome for CTPS cytoophidium. In order to reduce the background and contamination generated during sample preparation for MS analysis, and considering that ovary serves as a classical research tool in *Drosophila*, we decided to use ovaries as models to identify the proteome of CTPS cytoophidium, rather than using whole flies.

Next, we explored the biotinylation of normal and disrupted CTPS cytoophidia mediated by TbID in ovaries. We expressed CTPS-TbID and CTPS^H355A^-TbID via da-GAL4 driver and dissected adult flies, grown on either biotin-containing food or regular food since the early embryo stages. Using streptavidin-HRP blotting, we further confirmed that endogenous proteins of CTPS and CTPS^H355A^ were biotinylated by TbID (Figure 3C). In addition, immunostaining results revealed a normal expression for the wild-type and mutant CTPS in follicle cells (Figure 3D). Biotinylated proteins were detected using streptavidin-cy3 and immunofluorescence analysis showed extensive labeling signals for TbID, in the presence of biotin. Furthermore, we found that the biotinylated proteins had similar distribution patterns to the wild-type, or disrupted cytoophidia, which suggested that the proximate proteins of CTPS-TbID/CTPS^H355A^-TbID were easily biotinylated (Figure 3D). In the absence of exogenous biotin, some weak labeling signals were also detected by streptavidin-cy3 (Figure 3D), which was expected because TbID has great efficiency and consumes endogenous biotin in cells to function, as was reported in previous studies (Branon *et al.* 2018; Larochelle *et al.* 2019).

### A proof of principle study: cytoophidium proteome

**Figure 4:**
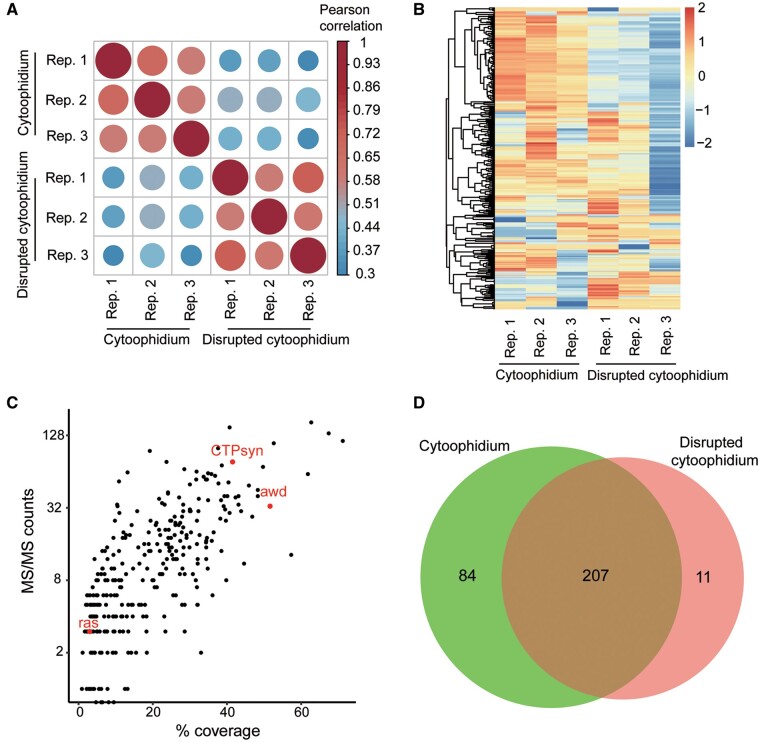
Proximate proteomes of wild-type and mutant CTPS in *Drosophila*. CTPS-TbID and CTPS^H355A^-TbID were expressed by da-GAL4 driver. About 60 ovaries from adult flies grown on biotin containing food were collected and prepared for MS assay. Each experiment was repeated three times. (A) Pearson correlation coefficients between replicate (Rep.) MS for cytoophidium and disrupted cytoophidium groups. (B) Hierarchical clustering of the proteome of CTPS cytoophidium and disrupted cytoophidium in three replicates. (C) Scatter plots by MS/MS counts up the *y*-axis and percentage sequence coverage (amino acids) on the *x*-axis. Points corresponding to previously established CTPS interacting proteins in STRING database are labeled in red. (D) Venn diagram showing overlap and unique enriched proteins adjacent to CTPS cytoophidium and disrupted cytoophidium.

To characterize the proteome of CTPS cytoophidium dependent on TbID biotinylation in *Drosophila*, we expressed CTPS-TbID and CTPS^H355A^-TbID ubiquitously by da-GAL4 driver. Then, about 60 ovaries from 14-day-old flies raised on biotin-containing food were collected, ground, and lysed. Biotinylated proteins were captured with streptavidin beads and subjected to on-bead trypsin digestion to generate peptides for analysis by MS. To assess the relative abundance of the characterized proteins, we utilized a label-free intensity-based quantification (LFQ) approach (Schwanhäusser *et al.* 2011) which was widely used in MS data analysis. Results showed that the three biological replicates for each group demonstrated good reproducibility, and showed a good correlation (Figure 4A).

Then, we analyzed the biotinylated proteins adjacent to CTPS-TbID or CTPS^H355A^-TbID by hierarchical clustering, and our results revealed the differences among the proteomes between normal CTPS cytoophidium and disrupted cytoophidium groups (Figure 4B). To assess the relative abundance of the characterized proteins, we analyzed MS/MS counts plotted against the protein sequence coverage (percentage of amino acid of a protein characterized by MS) by calculating the counts of all peptides matching to a specific protein (Larochelle *et al.* 2019; Schwanhäusser *et al.* 2011). In addition to CTPS, another two known CTPS-interacting proteins (Awd, Ras) were found in our assay, and, as expected, CTPS was determined as a top hit (Figure 4C). Ras (Enzyme name: Inosine monophosphate dehydrogenase) and CTPS, two cytoophidia forming metabolic enzymes, functions in rate‐limiting steps in the *de novo* synthesis of purine and pyrimidine nucleotides, respectively. IMPDH- and CTPS-based cytoophidia are aligned or intertwined in mammalian cells by using super-resolution confocal imaging (Chang *et al.* 2018). *Awd* (abnormal wing discs) encodes a nucleotide diphosphate kinase, which catalyzes the synthesis of nucleoside triphosphates other than ATP (FlyBase, last accessed on Mar.19 2021). These two enzymes and CTPS all function in nucleotides synthesis and their adjacency may coordinate their functions.

**Figure 5:**
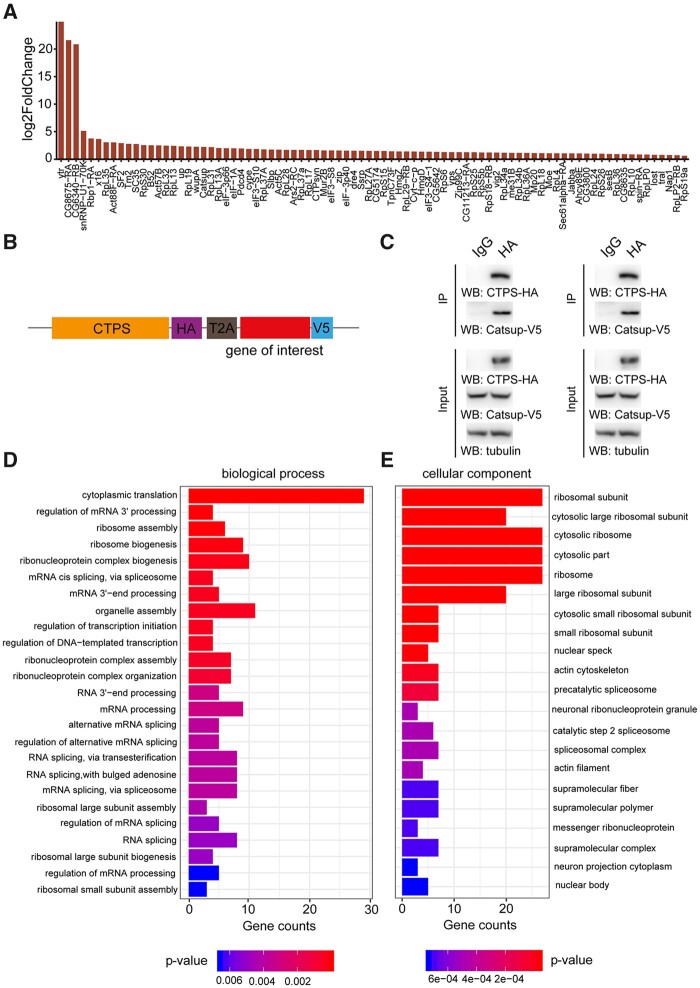
Validation and GO analysis of CTPS proximate proteomes. (A) Bar blot list of 84 enriched neighboring proteins of CTPS cytoophidium compared to disrupted cytoophidium. (B) Diagram of the expression cassettes used for Co-Immunoprecipitation (Co-IP) assays. T2A peptide mediates the expression of multiple transgenes containing HA or V5 tag in *Drosophila* S2 cells. (C) Co-IP of HA-tagged CTPS with V5-tagged Catsup and Pdcd4. Transfected S2 cells were lysed and immunoprecipitated with anti-HA magnetic beads or IgG bound protein A/G magnetic beads equally. The precipitates produced were examined by immunoblotting using anti-V5 antibody for Catsup and Pdcd4. (D) Enriched proximate proteins of CTPS cytoophidium classification based on BP. The single-item enrichment of *P*-value lower than 0.01 is shown and ranked by the *P*-value. (E) Enriched proximate proteins class distribution based on cellular components. *P*-value lower than 0.001 is shown.

By differential expression analysis of the biotinylated proteins in two groups, we found that there were 207 proteins that overlapped, while 84 proteins were enriched in the vicinity of cytoophidium location (Supplementary Table S1). However, 11 proteins resided adjacently to the disrupted cytoophidium (Figure 4D, Supplementary Figure S2). The enrichment of each protein, ranked by their fold change, is presented in Figure 5A. Ytr (yantar), CG8675, and CG6340 were highly enriched in CTPS cytoophidium proximate proteomes. Ytr functions in alternative splicing, while the MFs of CG8675 and CG6340 are unknown. Therefore, it remained undefined whether these alternative splicing elements or these unknown proteins functions in CTPS cytoophidium assembly.

To gain insights into PPI in CTPS cytoophidium proteome, co-immunoprecipitation assays were performed to verify the identified proteins that interact with CTPS. Because of the high noise background in MS, we focus on several proteins involved in enzymic regulation, cell survival, and organelle assembly. We choose some identified proteins for further validation in *Drosophila* S2 cells (Figure 5B). We found that CTPS-HA specifically immunoprecipitated Catsup-V5 and Pdcd4-V5 (Figure 5C). Catsup exhibits enzyme regulator activity and negatively regulates tyrosine hydroxylase activity (Stathakis *et al.* 1999). Pdcd4 functions in inhibition of translation and induction of apoptosis (Dikshit *et al.* 2013). Their vicinity of CTPS cytoophidia may facilitate their specific functions in certain subcellular localization or maintain their protein level and avoid degradation. In, addition, we found that the subunits eIF-3p66, eIF3-S8, eIF3-S10, eIF-3p40 of the eukaryotic translation initiation factor 3 (eIF3), are enriched in the CTPS cytoophidium vicinity. Subunits of eIF2/2B complexes have been found to form filamentous structures in budding yeast (Noree *et al.* 2010; Shen *et al.* 2016). Our results raised the question of whether the subunits of eIF3, as the orthologs of eIF2/2B, form filamentous structures in *Drosophila* or whether they affect the assembly of CTPS cytoophidium. This gives us a direction toward a comprehensive investigation of intracellular compartments of metabolic enzymes and their proximate proteome.

Subsequently, we further categorized the enriched proteins of CTPS cytoophidium after performing gene ontology (GO) analysis (Figure 5D-E). Several groups were overrepresented based on their biological process, including cytoplasmic translation, ribosome assembly, organelle assembly, ribonucleoprotein complex assembly, and ribosomal large subunit assembly (Figure 5D). In addition, GO analysis revealed significant enrichment of cellular components related to the ribosomal subunit, cytosolic part, actin cytoskeleton, actin filament, supramolecular fiber, supramolecular polymer, and supramolecular complex (Figure 5E). A previous study showed that CTPS functionally interacts with the intermediate filament, crescentin (CreS) to regulate cellular curvature in bacteria (Ingerson-Mahar *et al.* 2010). Here, we identified some proteins, such as Act57B and Act5C, which serve as components of supramolecular fiber/polymer or polymeric cytoskeletal fiber. Whether Act57B and Act5C are involved in the assembly of CTPS cytoophidium or they cooperate with CTPS to regulate cellular homeostasis has never been shown in *Drosophila*. Furthermore, enriched molecular functions included major clusters, such as the ones related to mRNA binding, actin binding, and cytoskeletal protein binding (Supplementary Figure S3). In our study, we characterized the proteome of CTPS cytoophidium, providing a reference for future exploration of potential cellular functions of CTPS compartmentation, coordinated with its neighboring proteins.

## Discussion

In order to systematically apply TurboID or miniTurbo to characterize the neighboring proteins of CTPS cytoophidium in *Drosophila*, we initially detected the conformational changes of CTPS filamentous structure by tagging TurboID or miniTurbo at its C-terminal, and we found that miniTurbo disrupted the normal structure of CTPS cytoophidium in *Drosophila* cultured cells (Figure 2A), probably as a result of the instability of miniTurbo. Thereby, we applied TurboID as the proximity ligase to probe neighboring proteins of CTPS cytoophidium in our study. First, our results revealed that TurboID can label the proteins in the vicinity of CTPS in a wide variety of tissues obtained from multiple developmental stages in *Drosophila* (Figure 2B and C). Meanwhile, we successfully demonstrated TurboID-mediated biotinylation in desired cells using a cell-type-specific gal4 driver in flies (Figure 2D).

To characterize the proteome of CTPS cytoophidium in *Drosophila*, a stringent control group is essential to be set out. The ideal approach is to disrupt long and curved filamentous structures of CTPS by point mutation. Previous studies reported that the single histidine mutation H355A on the tetramerization interface of human and mouse CTPS interferes with cytoophidia assembly *in vitro* and *in vivo* (Lynch *et al.* 2017; Sun and Liu 2019a). Here, we found that the amino acid histidine H355 is conserved among *Drosophila*, human and mouse (Figure 3A, Supplementary Figure S4) and that the mutant H355A impedes CTPS cytoophidium formation in *Drosophila* (Figure 3B). Thus, the disrupted filament induced by H355A was utilized as a parallel control in this study.

Using the TurboID-mediated biotinylation system coupled with MS, we recovered some previously reported proteins interacting with CTPS and identified 84 proteins enriched adjacently to CTPS cytoophidium, as compared to the case of disrupted cytoophidium (Figures 4D and 5A). Some factors, such as steric hindrance or the distance between CTPS and the proteins beyond the labeling radius of TurboID, could prevent the capture of other known CTPS-interacting proteins. Previous promiscuous ligases enable biotinylation of proximate proteins within a radius of approximately 10–20 nm (Gingras *et al.* 2019), and to some extent, the labeling radius depends on the linker length between ligases and bait proteins (Liu *et al.* 2018). In future studies of different bait proteins and subcellular organelles, the linker length should be considered for efficient labeling. We mapped the enriched proteins in the vicinity of CTPS cytoophidia, and found that they are involved in different BP and are important constituents of cellular/subcellular organelles (Figure 5D and E). In the future, the regulatory aspects of the relationship between CTPS cytoophidium and its proximate proteins need to be examined in detail.

In summary, we have addressed all these questions asked previously and demonstrated the feasibility of TurboID-mediated labeling method in a wide range of developmental stages, tissues, and specific cells in *Drosophila*. We also utilized this method, as a proof-of-principle, for studying the proteome of CTPS cytoophidia and identified several interactors. TurboID-mediated proximity labeling system provides a possible solution to explore the proximate proteins and cellular functions of subcellular compartments of metabolic enzymes *in vivo*.
