# Correction to: Highly effective proximate labeling in *Drosophila*

**DOI:** 10.1093/g3journal/jkac005

**Published:** 2022-01-28

**Authors:** Bo Zhang, Yuanbing Zhang, Ji-Long Liu

*G3 Genes|Genomes|Genetics*, 2021, 11(5), jkab077 https://doi.org/10.1093/g3journal/jkab077

When this paper first published online, there was an error in figure 5. Figure 5 originally appeared as

**Figure 5:**
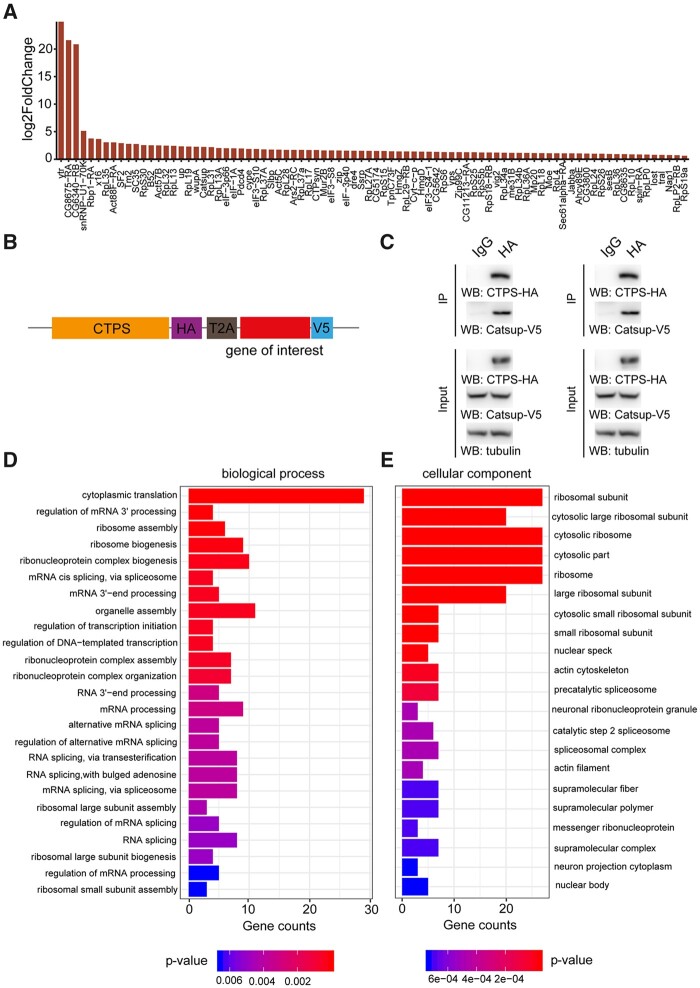
Validation and GO analysis of CTPS proximate proteomes. (A) Bar blot list of 84 enriched neighboring proteins of CTPS cytoophidium compared to disrupted cytoophidium. (B) Diagram of the expression cassettes used for Co-Immunoprecipitation (Co-IP) assays. T2A peptide mediates the expression of multiple transgenes containing HA or V5 tag in *Drosophila* S2 cells. (C) Co-IP of HA-tagged CTPS with V5-tagged Catsup and Pdcd4. Transfected S2 cells were lysed and immunoprecipitated with anti-HA magnetic beads or IgG bound protein A/G magnetic beads equally. The precipitates produced were examined by immunoblotting using anti-V5 antibody for Catsup and Pdcd4. (D) Enriched proximate proteins of CTPS cytoophidium classification based on BP. The single-item enrichment of *P*-value lower than 0.01 is shown and ranked by the *P*-value. (E) Enriched proximate proteins class distribution based on cellular components. *P*-value lower than 0.001 is shown.

**Figure 5:**
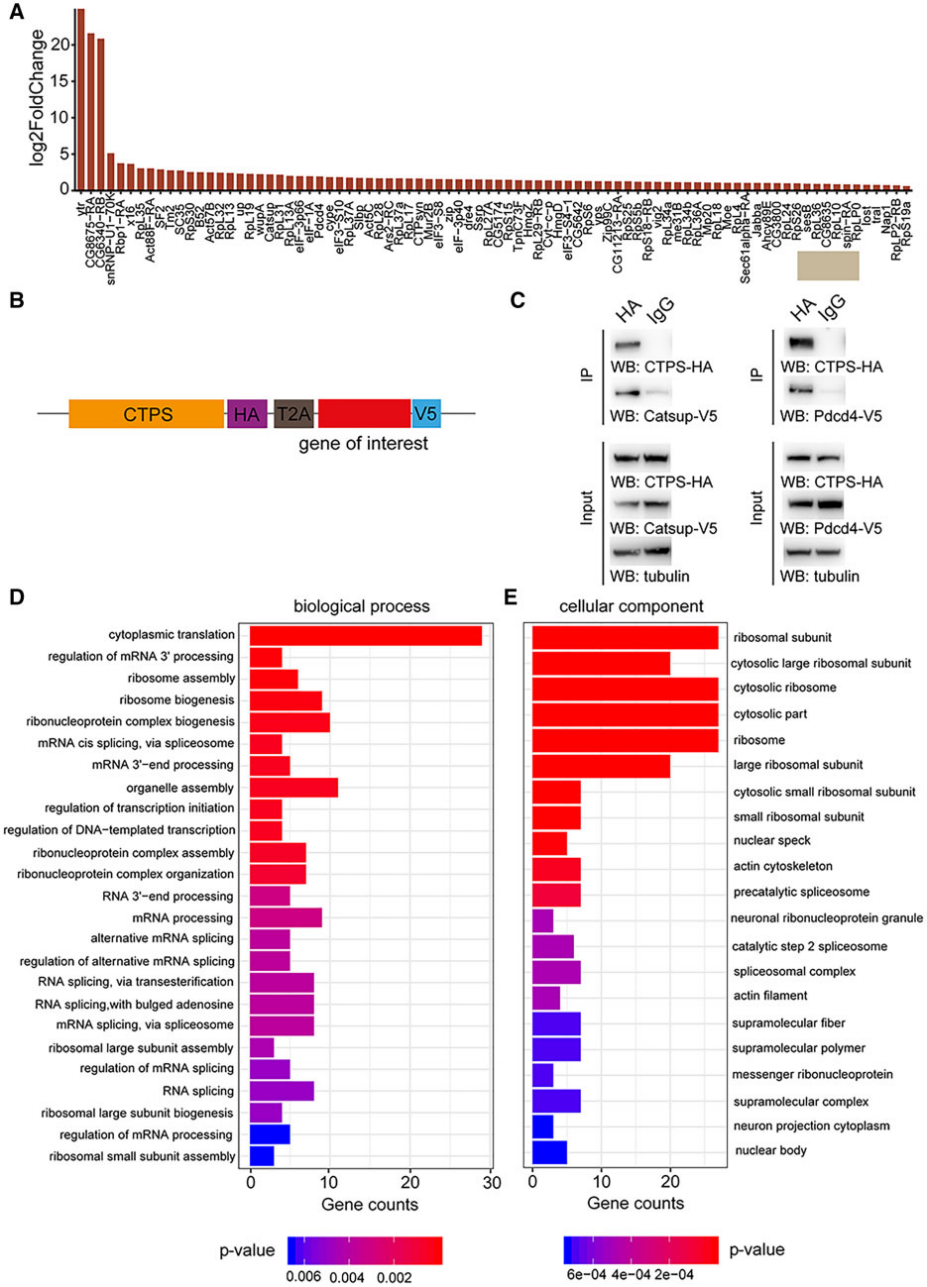
Validation and GO analysis of CTPS proximate proteomes. (A) Bar blot list of 84 enriched neighboring proteins of CTPS cytoophidium compared to disrupted cytoophidium. (B) Diagram of the expression cassettes used for Co-Immunoprecipitation (Co-IP) assays. T2A peptide mediates the expression of multiple transgenes containing HA or V5 tag in *Drosophila* S2 cells. (C) Co-IP of HA-tagged CTPS with V5-tagged Catsup and Pdcd4. Transfected S2 cells were lysed and immunoprecipitated with anti-HA magnetic beads or IgG bound protein A/G magnetic beads equally. The precipitates produced were examined by immunoblotting using anti-V5 antibody for Catsup and Pdcd4. (D) Enriched proximate proteins of CTPS cytoophidium classification based on BP. The single-item enrichment of *P*-value lower than 0.01 is shown and ranked by the *P*-value. (E) Enriched proximate proteins class distribution based on cellular components. *P*-value lower than 0.001 is shown.

Figure 5 should have appeared as

